# Editorial: Exploring human resources in the context of projects

**DOI:** 10.3389/fpsyg.2023.1166597

**Published:** 2023-06-14

**Authors:** Tomas Blomquist, Ali Farashah

**Affiliations:** ^1^Umeå School of Business, Economics and Statistics, Umeå University, Umeå, Sweden; ^2^Management & Organization Department, School of Business, Society and Engineering, Mälardalen University, Västerås, Sweden

**Keywords:** project, human resource (HR) management, temporal organization, organizational behavior, project organization

Projects make up a large part of our working life. We see construction workers work on one construction site and move to a new one as soon as the building, road, or bridge is done. Researchers move from one project to the other as research grants are coming. HR and controllers take care of staffing, and budget tasks as projects and other temporal activities evolve and finalize. Projects require integration and coordination between the project and the organization, resulting in the dual embeddedness of project resources and employees within the project and the organizational setting (Sydow et al., 2004). The uncertainty, urgency, and integration tied with the project make the management of people in temporal organizations different than the traditional organizations where operations are more routine and repetitive.

Following Geraldi and Söderlund (2018), this Research Topic is a small response and contribution to the call for more project studies and the need to expand the scope from a single project to portfolios and networks and to include a new perspective on studies of work-life in projects and temporal organizations. This Research Topic is inspired by various scholars in organizational psychology, human resource management, and project management to bridge the gap in the fields by looking at areas such as project manager career paths (Akkermans et al., 2020); recruitment and project management certification (Farashah et al., 2019); employment (Storey et al., 2002; Raja et al., 2013); psychological contracts (Scheel et al., 2013) and, the role of HRM in projects (Söderlund and Bredin, 2006; Welch et al., 2008). The list could be longer, and the articles included show the importance of going beyond traditional project management studies and moving forward into project studies.

Samimi uses a comparative case study in the Scottish oil and gas industry and takes a practice-oriented approach to clarify how HRM practices are being shaped and transformed in a project-based context. Findings reveal that project characteristics, specifically their duration, size, and technical properties, induce different temporalities, reflected in the pace of practices, and, along with different work locations and inter-organizational relationships, impact HRM practices. The article is one example of project studies beyond the traditional project management studies of project success factors.

Yao et al. studied the influence of low-price winning bids on the probability of unsafe work behavior of construction workers. Using an on-site questionnaire on subcontract management, low-cost bid winning, and construction experiences. Data analyses show that winning bids at lower prices had a weak effect on workers' intentions to behave unsafely. However, subcontractor management significantly impacts workers' intentions for unsafe behavior on the job site. They also show that winning a bid at a low price indirectly affects the safety management of the subcontractor as a whole and indirectly affects the willingness of site workers to act unsafely.

Zhang et al. explore the influence of failure aversion on project failures. Sensemaking theory was used to understand individual error avoidance and how it affects learning from errors through reasoning and expectation. They showed that in the context of the Chinese R&D teams, individuals' failure aversion enhanced their learning from failure by inducing a loss-focused coping. Still, failure aversion negatively affected learning from failure by increasing the individuals' perceived loss of self-esteem. The study contributes to the literature both learning on the individual level and the underlying cognitive mechanisms operating between failure aversion and learning from project failure.

Naseer et al. demonstrated that the project team characteristics (e.g., cohesive and experienced members) and project manager characteristics (e.g., experience, managerial skills, and PM technical skills) are more critical success factors than design-related characteristics such as project complexity and planning specificity. The effects of culture on knowledge creation in the context of Pakistan as a collectivist and developing country are also elaborated.

Liu et al. investigated the process of knowledge transfer in university-industry collaboration enacted as postdoctoral projects in Chinese firms (i.e., postdoctoral workstations). Having a quasi-natural experiment, the study expands the existing research primarily focusing on the postdoc individuals by including factors such as career intentions and the challenges they face, this study examined human resource development strategy and governance of labor investment decisions. The postdoctoral workstation increases efficiency in investment in human capital and performance. Knowledge as an implicit resource enhances organizational capacity often in the long term, and this can have implications for measuring project success in project-oriented organizations. In the case of knowledge creation projects such as postdoctoral workstations, R&D projects, and co-creation projects the traditional cost, time, and scope triangle for measuring project success might need to be revised.

## Author contributions

TB and AF invited potential authors to this Research Topic and managed the review process. All authors contributed to the article and approved the submitted version.
